# The biomarkers related to immune infiltration to predict distant metastasis in breast cancer patients

**DOI:** 10.3389/fgene.2023.1105689

**Published:** 2023-02-22

**Authors:** Chengsi Ren, Anran Gao, Chengshi Fu, Xiangyun Teng, Jianzhang Wang, Shaofang Lu, Jiahui Gao, Jinfeng Huang, Dongdong Liu, Jianhua Xu

**Affiliations:** ^1^ Department of Laboratory Medicine, Shunde Hospital of Guangzhou University of Chinese Medicine, Foshan, China; ^2^ Department of Pathology, Shunde Hospital of Guangzhou University of Chinese Medicine, Foshan, China; ^3^ Department of Laboratory Science, The Second Affiliated Hospital of Guangzhou University of Chinese Medicine, Guangzhou, China

**Keywords:** distant metastasis, gene signature, nomogram, immune infiltration, myeloid cells

## Abstract

**Background:** The development of distant metastasis (DM) results in poor prognosis of breast cancer (BC) patients, however, it is difficult to predict the risk of distant metastasis.

**Methods:** Differentially expressed genes (DEGs) were screened out using GSE184717 and GSE183947. GSE20685 were randomly assigned to the training and the internal validation cohort. A signature was developed according to the results of univariate and multivariate Cox regression analysis, which was validated by using internal and external (GSE6532) validation cohort. Gene set enrichment analysis (GSEA) was used for functional analysis. Finally, a nomogram was constructed and calibration curves and concordance index (C-index) were compiled to determine predictive and discriminatory capacity. The clinical benefit of this nomogram was revealed by decision curve analysis (DCA). Finally, we explored the relationships between candidate genes and immune cell infiltration, and the possible mechanism.

**Results:** A signature containing CD74 and TSPAN7 was developed according to the results of univariate and multivariate Cox regression analysis, which was validated by using internal and external (GSE6532) validation cohort. Mechanistically, the signature reflect the overall level of immune infiltration in tissues, especially myeloid immune cells. The expression of CD74 and TSPAN7 is heterogeneous, and the overexpression is positively correlated with the infiltration of myeloid immune cells. CD74 is mainly derived from myeloid immune cells and do not affect the proportion of CD8+T cells. Low expression levels of TSPAN7 is mainly caused by methylation modification in BC cells. This signature could act as an independent predictive factor in patients with BC (*p* = 0.01, HR = 0.63), and it has been validated in internal (*p* = 0.023, HR = 0.58) and external (*p* = 0.0065, HR = 0.67) cohort. Finally, we constructed an individualized prediction nomogram based on our signature. The model showed good discrimination in training, internal and external cohort, with a C-index of 0.742, 0.801, 0.695 respectively, and good calibration. DCA demonstrated that the prediction nomogram was clinically useful.

**Conclusion:** A new immune infiltration related signature developed for predicting metastatic risk will improve the treatment and management of BC patients.

## Introduction

Currently, breast cancer (BC) has been the highest cause of cancer death for women (Sung et al., 2021)^.^ Approximately 90% of patient death come from metastatic complications although only 20%–30% of patients suffer from metastatic recurrence, which makes the treatment schedules of metastatic patients different from those of non-metastatic patients (Chen et al., 2018; Medeiros and Allan, 2019)^.^ Therefore, accurate identification of distant metastasis (DM) in each individual patient with BC is crucial for determining individualized follow-up strategy and the optimal treatment regimen. The further exploration of new biomarkers is of great significance.

BC is a heterogeneous disease at molecular levels, which is associated with distinct patterns of metastatic spread (Liang et al., 2020)^.^ As a result of different molecular subtypes, accurate prediction of DM is still a great challenge. Notably, gene expression-based molecular subtyping appears to have clinical implications for the treatment of patients with BC (Masuda et al., 2013; Prat et al., 2015). Moreover, previous studies have shown that molecular subtypes of BC could be considered as a risk factor for distant recurrence (Gnant et al., 2014; Tobin et al., 2017), This suggests that gene expression profiling has a great value in predicting the probability of DM (Ellis et al., 2011; Filipits et al., 2014). However, what pattern of gene expression causes this difference still remains unclear. In this study, we aim to find gene biomarker associated with metastasis and construct a gene signature that could accurately predict distant metastasis–free survival (DMFS).

Nomogram, a simple devices for predicting the likelihood of disease, is widely used in the field of oncology (Balachandran et al., 2015). However, there is no literature that has applied gene signature to a nomogram for predicting DMFS in BC, although multiple predictive nomograms have been constructed for patients with BC (Rouzier et al., 2005; Wang et al., 2019; Huang et al., 2020). Therefore, the aim of this study was to provides a reliable nomogram to predict the risk of metastasis development based on gene signature in patients with BC.

## Materials and methods

### Data collection and pre-processing

Data of the cancer and adjacent normal tissues of samples with metastasis were downloaded from the Gene Expression Omnibus (GEO) database: GSE183947 and GSE184717. Meanwhile, GSE20685 and GSE6532 were singled out to construct a gene signature and a nomogram. Samples without complete clinical information or based on different platforms were regarded as substandard samples in the present study. Subsequently, batch effects were removed using Combat from R package SVA (Johnson et al., 2007).

### Acquisition of differentially expressed genes (DEGs)

An R package limma (Ritchie et al., 2015) was applied to identify DEGs between cancer and adjacent normal tissues. The threshold was set to |logFC| >2 and the adjusted *p* < 0.05. Then, we use Venn diagrams to find the intersection of DEGs that simultaneously upregulated or downregulated in both metastatic tumor and primary tumor.

### Screening of optimal predictive biomarkers and development of signature

GSE20685 was randomized 1:1 and split into a training cohort and an internal validation cohort. For reproducibility, the random seed was used and set to 3. To find optimal predictive biomarkers, univariate and multivariate Cox regression analysis was performed in the training cohort with a *p*-value cutoff of 0.05. Then, the regression coefficient was defined according to the multivariate Cox regression model and the formulas were described as follows:

$$risk\;score=\overset{N}{\underset{i=1}{\sum }}\left(Exp_{i}*Coe_{i}\right)$$

Finally, results were visualized using the “forestplot” package in R.

### Construction and assessment of the nomogram

In order to make better use of the signature, a nomogram was constructed using the “RMS” package. The Harrell’s Concordance index (C-index) values range from 0 to 1, which is positively correlated to the predictive performance of the nomogram (Harrell et al., 1996). The nomogram was subjected to bootstrapping validation (1,000 bootstrap resamples) to calculate a relatively corrected C-index. To assess the consistency of DMFS at 3-, 5-, and 10-year between the nomogram predicted probabilities and observed rates, calibration curves were plotted.

### Evaluation of predictive value

Gene Expression Profiling Interactive Analysis (GEPIA) (http://gepia.cancer-pku.cn/) is an online tool, which contains massive RNA sequencing data from The Cancer Genome Atlas (TCGA) and Genotype-Tissue Expression (GETx) (Tang et al., 2017). We analyze the differential gene expression and correlation between BC tissues (*n* = 1,085) and normal tissues (*n* = 291) using GEPIA.

In terms of the signature, the optimal cutoff value was calculated according to the median value of the signature in the training cohort. Then we use it to divide patients into two groups and predict the DMFS in the training, internal validation, and external validation cohort by means of plotting survival curves based on the Kaplan-Meier method. To date fifty-five dataset have been included in Breast Cancer Gene-Expression Miner v4.7 (bc-GenExMiner) (http://bcgenex.centregauducheau.fr/), which can be used to improve gene prognostic analysis performance (Jézéquel et al., 2012). Candidate biomarkers were validated again using bc-GenExMiner.

Compared with ROC curves, decision curve analysis (DCA) can integrate patient and doctor preference into analysis, which is increasingly being utilized in clinical practice (Huang et al., 2016). To evaluate the clinical utility of models, DCA curves were developed using the “stdca.R” package in R.

### Gene set enrichment analysis

The degree of differential gene expression was reordered by high- and low-score groups instead of definite differential gene thresholds, which was used to screen significantly enriched KEGG pathways. This method helps minimize losses of original gene expression data (Subramanian et al., 2005). We performed Gene set enrichment analysis (GSEA) to analyze the difference in anti-metastatic potential between two groups.

### Immune infiltration analysis

The relationship between optimal predictive biomarkers and immune infiltrating levels was analyzed using Timer (https://cistrome.shinyapps.io/timer/) (Li et al., 2017). An algorithm named quanTIseq was used to estimate the fraction of immune cell subsets infiltrating the tissue from GSE20685 and GSE6532 (Finotello et al., 2019). Student’s t-test was used to test for significance between high-score group and low-score group.

### Immunohistochemistry

28 tissue samples of BC were collected in Shunde Hospital of Guangzhou University of Chinese Medicine to analyze the correlation between CD74 and myeloid cells. To reduce error, Immunohistochemistry (IHC) for CD74 (1:200 dilution; EPR4064, Abcam) and CD33 (1:200 dilution; EPR23051-101, Abcam) were performed by an autostrainer system (Lumatas Titan, LumatasBiosystem Inc.) on 3-μm-thick,formalin-fixed and paraffin-embedded (FFPE) Sections. Counterstaining was done with hematoxylin. The average optical density (AOD) was calculated with Image Pro Plus6.0 to determine the protein expression level.

### Cell culture

BC cell line MCF-7,ZR-75-1, MDA-MB-231 and myeloid leukemia cell line K562 were all obtained from the Experimental Center, Shunde Hospital of Guangzhou University of Chinese Medicine (Foshan, China). Three breast cell lines were maintained in DMEM medium with 10% fetal bovine serum (FBS). Cells were seeded in 6-well plates and, 12 h after plating, demethylation was induced with 10 μM 5-aza for 24 h. Stable expression of TSPAN7 was confirmed by RT-qPCR and Western blot.

### qRT-PCR

Total RNA was extracted using TRIzol (Invitrogen) and a two-step reverse transcription-quantitative PCR (RT-qPCR) protocol was performed using PrimeScript RT Master Mix (Takara) and TB Green Premix Ex Taq (Takara), following manufacturer’s instructions. GAPDH was used as a loading control. The sequences of TSPAN7 primers were as follows: 5′-CTG​GCT​GTT​GGA​GTC​TGG-3′ (forward); 5′-CCG​ATG​AGC​ACA​TAG​GGA' (reverse). The sequences of GAPDH primers were:5′-CGGATTTGGTCGTATTGGG-3′ (forward); 5′-CTG​GAA​GAT​GGT​GAT​GGG​ATT-3′ (reverse). The experiment was repeated three times biologically for statistical analysis. The relative expression was quantified using the 2^−ΔΔCT^ method.

### Western blot analysis

Cells were placed on ice and lysed with RIPA buffer (Beyotime, China) containing protease inhibitors Cocktail (MCE,China). Proteins were resolved on SDS-PAGE gels, transferred to PVDF membranes (Millipore, United States), and incubated at 4°C overnight with TSPAN7 primary antibodies diluted at 1:1,000 (ProteinTech, 18695-1-AP) and β-tubulin loading control antibodies diluted at 1:1,000 (Servicebio, GB11017) and the secondary antibody diluted at 1:10,000 (Abcam,ab6721). Bands were visualized using an ECL kit.

## Results

### Screening of DEGs

**FIGURE 1:**
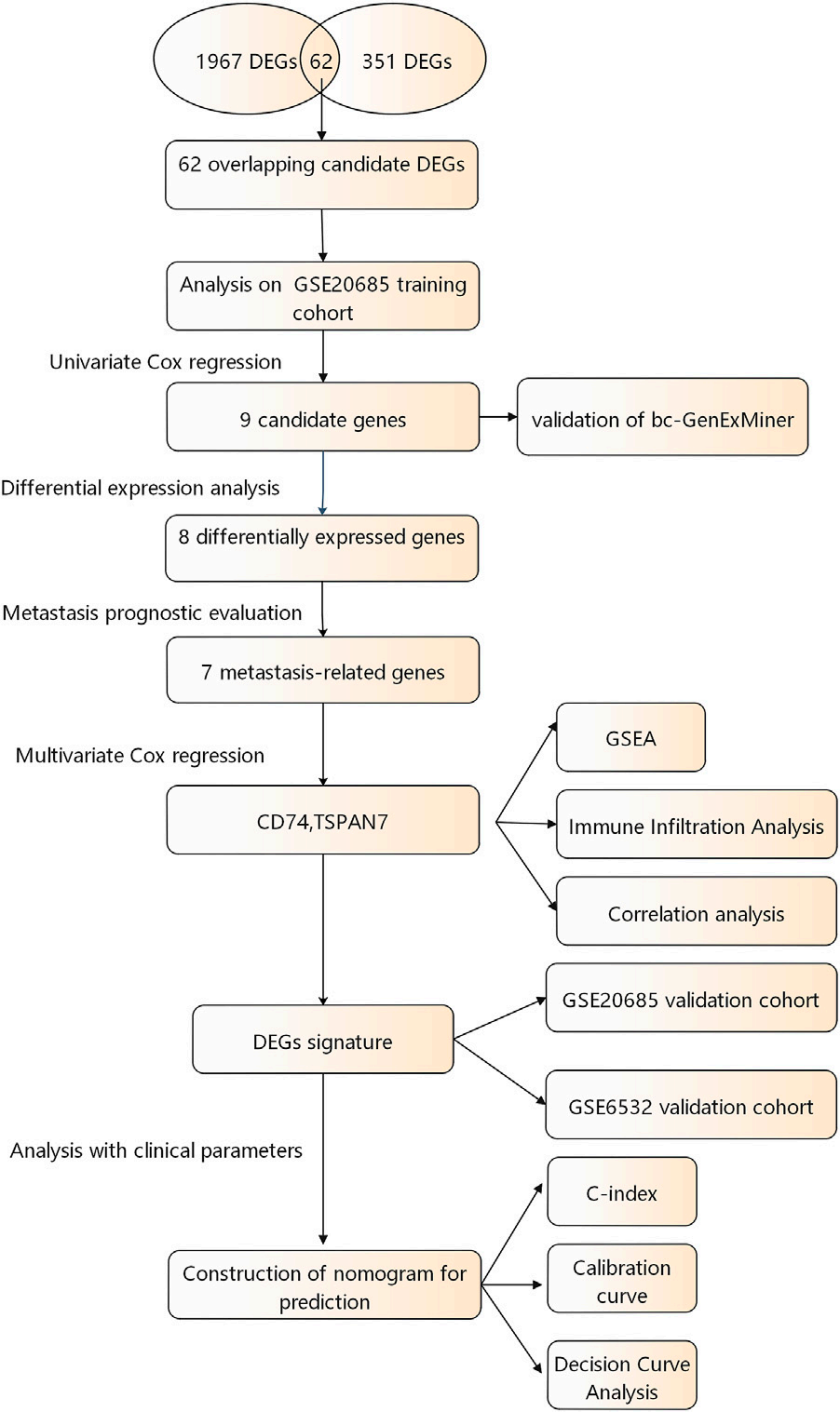
A flowchart of the study procedure.

**FIGURE 2:**
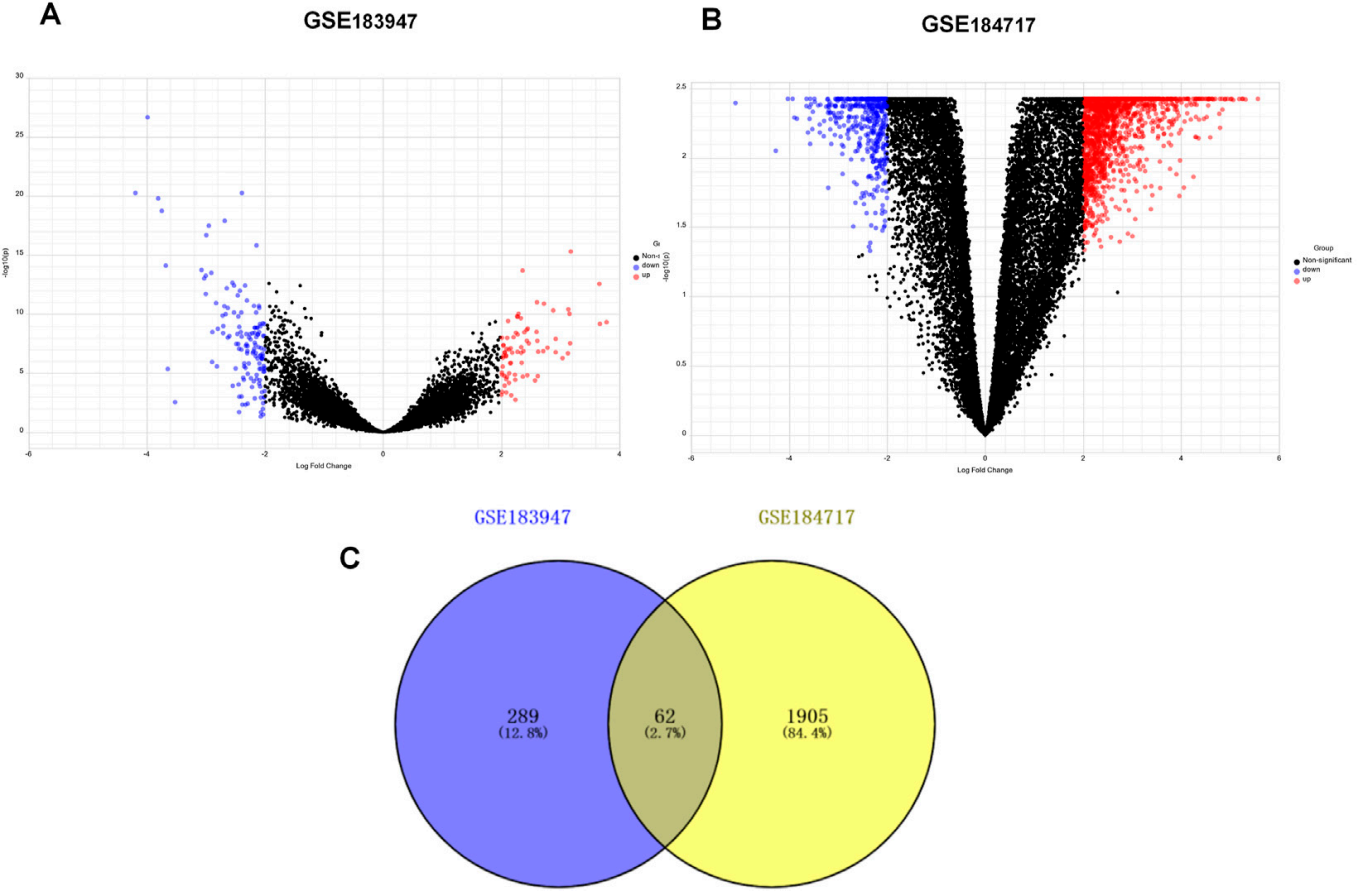
Identification of differentially expressed genes (DEGs). **(A)** Volcano plot of DEGs between primary tumor and paired normal tumor in GSE183947. **(B)** Volcano plot of DEGs between metastasis tumor and paired normal tumor in GSE184717. **(C)** Venn diagram showing the intersection of the DEGs in GSE 183947 and GSE 184717.

A flow chart of the study design is shown in Figure 1. The R package “limma” was used to screen DEGs between tumor and normal tissues in GSE184717 and GSE183947, where a total of 1967 (1,494 upregulated and 473 downregulated) and 351 (180 upregulated and 171downregulated) DEGs were obtained, respectively. The distribution of each gene was visualized by volcano plots (Figures 2A,B). The resulting list of DEGs was the intersection between the above datasets and a total of 62 overlapping DEGs were obtained (Figure 2C).

### Two genes were screened out as potential predictive biomarkers

**FIGURE 3:**
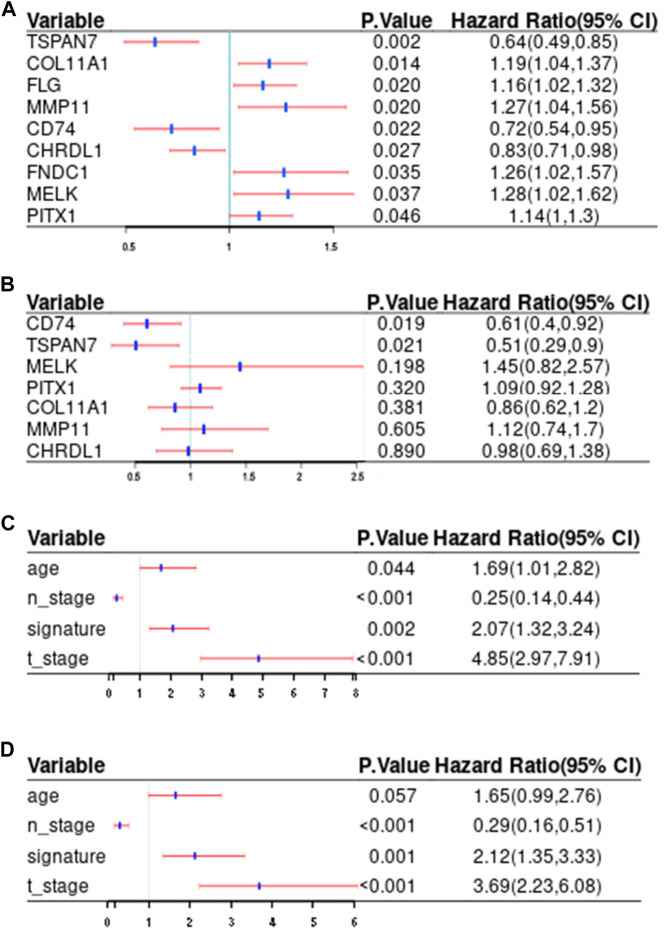
Screening of potential prediction biomarkers and risk factors for distant metastasis. **(A, B)** Univariate **(A)** and multivariate **(B)** Cox regression analysis of the metastasis-related genes. **(C, D)** Univariate **(C)** and multivariate **(D)** Cox regression analysis of correlations between the signature and clinical traits. Red and blue, respectively indicate 95% confidence interval and hazard ratio. T_stage, tumor size; N_stage, nodal status; Signature, the risk score.

The clinicopathologic characteristics are summarized in Table 1. To identify DEGs that are associated with metastasis, univariate Cox regression analysis was performed. A total of nine candidate genes (CD74, TSPAN7, COL11A1, FLG, MMP11, CHRDL1, FNDC1, MELK, PITX1) with an adjusted *p* < 0.05 were screened out (Figure 3A). Detailed information for each gene was listed in Table 2.

**TABLE 1:** The relationship between the signature and patient characteristics.

Variables	GSE20685 (Training cohort)			*p*-value	GSE20685 (validation cohort)			*p*-value	GSE6531 (validation cohort)			*p*-value
	Total (*n* = 164)	High risk (*n* = 82)	Low risk (*n* = 82)		Total (*n* = 163)	High risk (*n* = 79)	Low risk (*n* = 84)		Total (*n* = 87)	High risk (*n* = 41)	Low risk (*n* = 46)	
**Age(years)**												
≤60	143	70 (49%)	73 (51%)	0.483	139	67 (48%)	72 (52%)	0.869	36	11 (31%)	25 (69%)	0.009
>60	21	12 (57%)	9 (43%)		24	12 (50%)	12 (50%)		51	30 (59%)	21 (41%)	
**T stage**												
T1-T2	144	70 (49%)	74 (51%)	0.34	146	72 (49%)	74 (51%)	0.525	83	39 (47%)	44 (53%)	0.906
T3-T4	20	12 (60%)	8 (40%)		17	7 (41%)	10 (59%)		4	2 (50%)	2 (50%)	
**N stage**												
N0	63	34 (54%)	29 (46%)	0.422	74	35 (47%)	39 (53%)	0.786	29	17 (59%)	12 (41%)	0.129
N+	101	48 (48%)	53 (52%)		89	44 (49%)	45 (51%)		58	24 (41%)	34 (59%)	
**M stage**												
M0	103	44 (43%)	59 (57%)	0.015	141	63 (45%)	78 (55%)	0.014	59	22 (37%)	37 (63%)	0.008
M1	61	38 (62%)	23 (38%)		22	16 (73%)	6 (27%)		28	19 (68%)	9 (32%)	
**Expression of CD74**												
<mean	86	66 (77%)	28 (23%)	<0.001	79	59 (75%)	20 (25%)	<0.001	41	29 (71%)	12 (29%)	<0.001
≥mean	78	16 (21%)	62 (79%)		84	20 (24%)	64 (76%)		46	12 (26%)	34 (74%)	
**Expression of TSPAN7**												
<mean	75	62 (83%)	13 (17%)	<0.001	84	64 (76%)	20 (24%)	<0.001	49	37 (76%)	12 (24%)	<0.001
≥mean	89	20 (22%)	69 (78%)		79	15 (19%)	64 (81%)		38	4 (11%)	34 (89%)	

**TABLE 2:** Details of the candidate genes.

Gene Symbol	Gene Id	Full name	Cytogenetic locations	Protein function
**CD74**	972	CD74 molecule	5q33.1	CD markers; Disease related genes; Cancer-related genes: Candidate cancer biomarkers
**TSPAN7**	7,102	tetraspanin 7	Xp11.4	Transporters: Accessory Factors Involved in Transport; CD markers; Disease related genes; Potential drug targets
**COL11A1**	1,301	collagen type XI alpha 1 chain	1p21.1	Predicted intracellular proteins; Disease related genes; Predicted secreted proteins; Cancer-related genes: Candidate cancer biomarkers
**FLG**	2,312	filaggrin	1q21.3	Cancer-related genes: Mutated cancer genes; Predicted intracellular proteins; Disease related genes
**MMP11**	4,320	matrix metallopeptidase 11	22q11.23	Peptidases: Metallopeptidases; Cancer-related genes: Candidate cancer biomarkers; FDA approved drug targets: Small molecule drugs; Predicted secreted proteins; Enzymes
**CHRDL1**	91,851	chordin like 1	Xq23	Disease related genes; Predicted secreted proteins
**FNDC1**	84,624	fibronectin type III domain containing 1	6q25.3	Predicted intracellular proteins; Predicted secreted proteins
**MELK**	9,833	maternal embryonic leucine zipper kinase	9p13.2	Disease related genes; ENZYME proteins: Transferases; Predicted intracellular proteins; Potential drug targets; Kinases: CAMK Ser/Thr protein kinases; Enzymes
**PITX1**	5,307	paired like homeodomain 1	5q31.1	Predicted intracellular proteins; Disease related genes; Transcription factors: Helix-turn-helix domains

**FIGURE 4:**
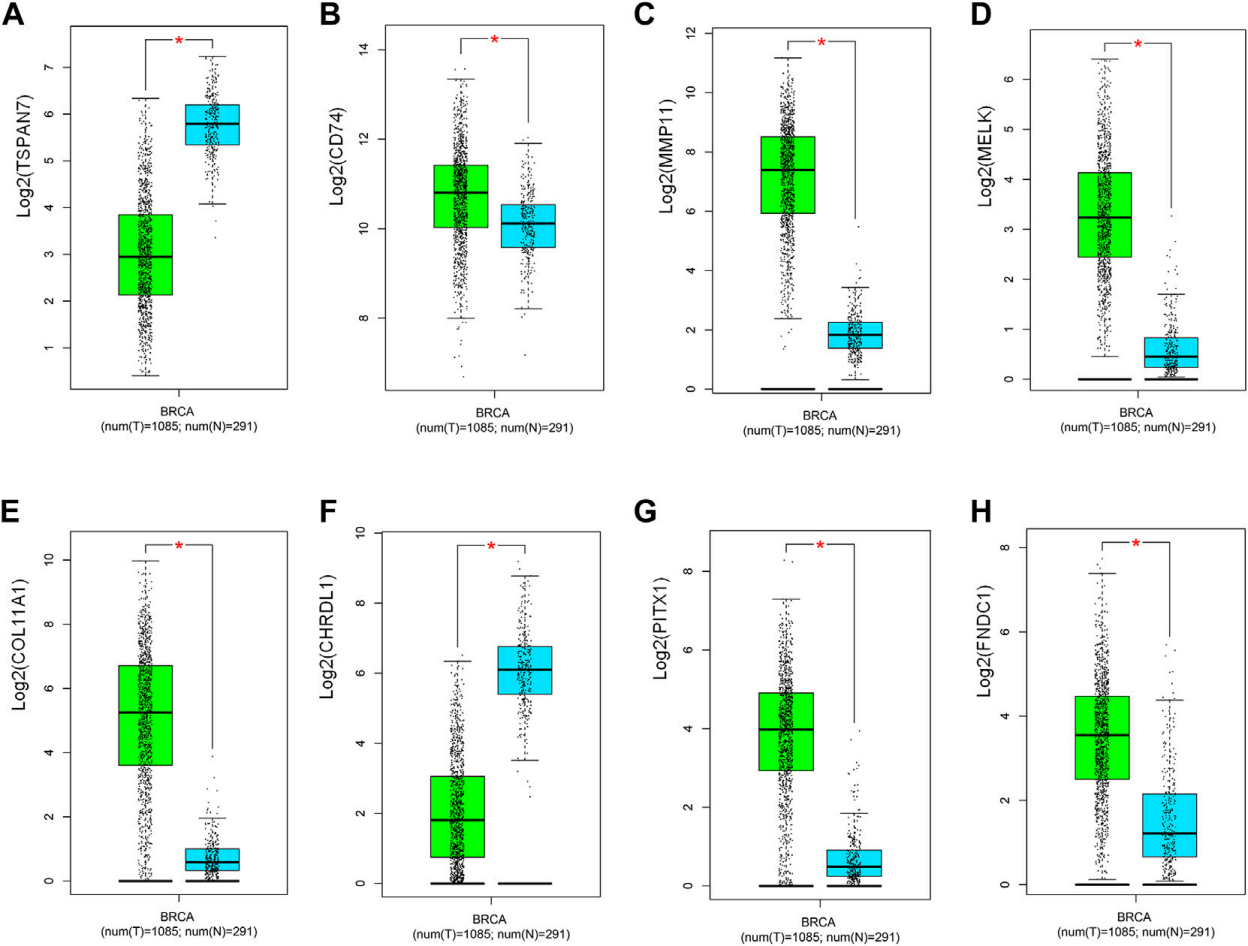
Validation of the expression of potential predictive biomarker in TCGA and GETx (*n* = 1,376). **(A)** TSPAN7, **(B)** CD74, **(C)** MMP11, **(D)** MELK, **(E)** COL11A1, **(F)** CHRDL1, **(G)** PITX1, **(H)** FNDC1. T: tumor; N: normal. Green and blue, respectively indicate tumor and normal groups. *p < 0.05.

We further analyzed the differential gene expression between BC tissues (*n* = 1,085) and normal tissues (*n* = 291) using GEPIA. As shown in Figure 4, eight genes were found to be differentially expressed in BC tissues, including six upregulated genes (CD74,MMP11,MELK,COL11A1,PITX1,FNDC1) and two downregulated genes (TSPAN7, CHRDL1).

Interestingly, breast tissues with low CD74 expression were related to poorer DMFS although CD74 commonly upregulated in BC patients.

**FIGURE 5:**
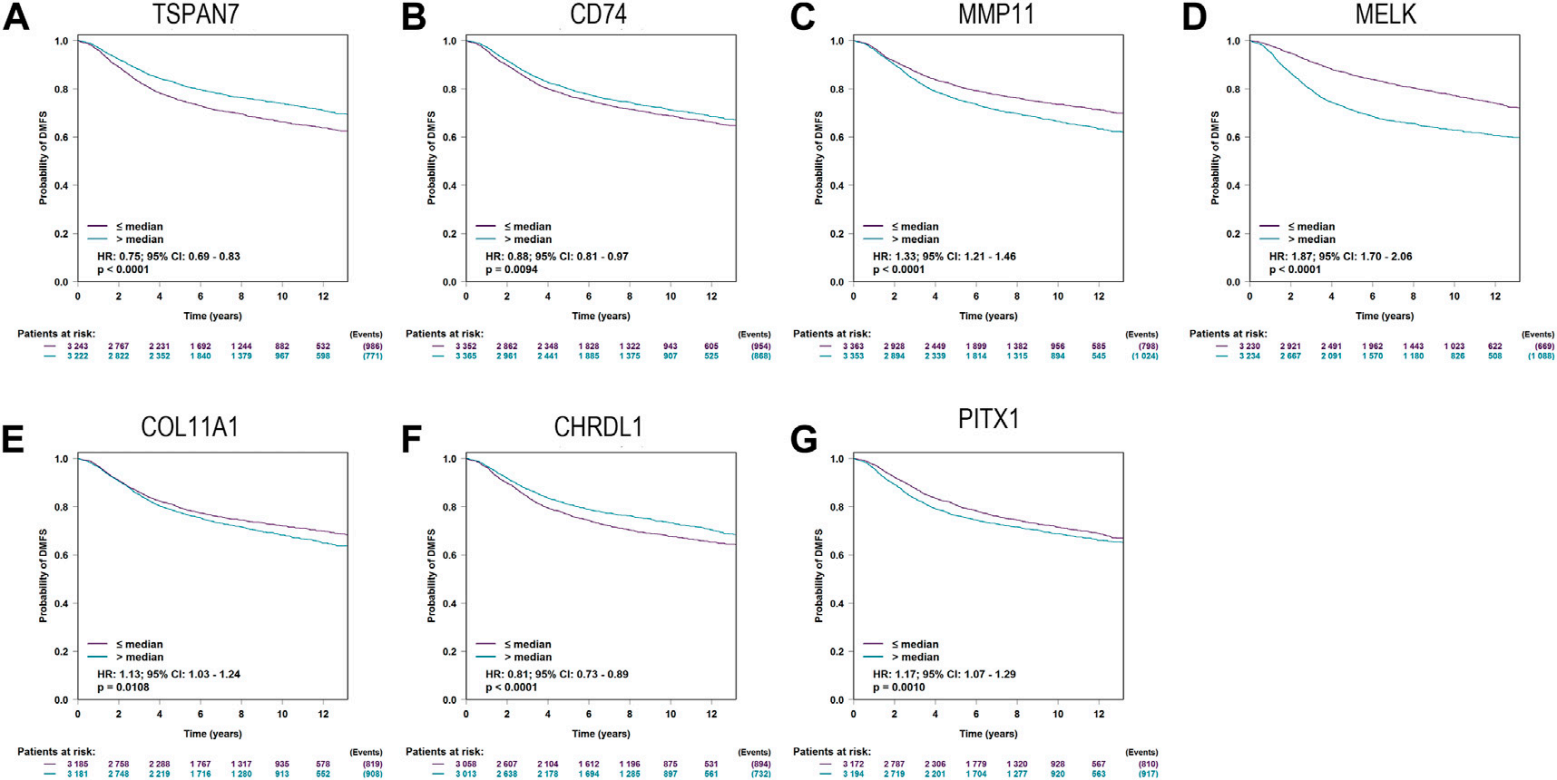
Kaplan-Meier curves for Distant metastasis-free survival (DMFS) of TSPAN7 **(A)**, CD74 **(B)**, MMP11 **(C)**, MELK **(D)**, COL11A1 **(E)**, CHRDL1 **(F)**, and PITX1 **(G)**in breast cancer patients using bc-GenExMiner.

Additionally, bc-GenExMiner was utilized to validate the relationship between predictive biomarkers and DMFS. The expression levels of seven genes, including TSPAN7(HR:0.75; 95%CI:0.69-0.83; *p* < 0.0001), CD74(HR:0.88; 95%CI:0.81-0.97; *p* = 0.0094), MMP11 (HR:1.33; 95%CI:1.21-1.46; *p* < 0.0001), MELK(HR:1.87; 95%CI:1.70-2.06; *p* < 0.0001), COL11A1(HR:1.13; 95%CI:1.03-1.24; *p* = 0.0108), CHRDL1(HR:0.81; 95%CI:0.73-0.89; *p* < 0.0001), PITX1(HR:1.17; 95%CI:1.07-1.29; *p* = 0.0010), were found able to estimate DMFS (Figure 5).

Finally, CD74 (HR:0.61; 95%CI:0.4-0.92; *p* = 0.019) and TSPAN7(HR:0.51; 95%CI:0.29-0.9; *p* = 0.021) were screened out to develop a signature according to the results of multivariate Cox analysis (Figure 3B).

### Development and validation of the signature

We calculated the signature based on the expression of CD74 and TSPAN7 as follows:

Signature = (0.7,697,433 X expression of CD74) + (0.6633031 X expression of TSPAN7).

Of note, the expression of CD74 and TSPAN7 was negatively correlated with DMFS, suggesting that patients with low scores have a higher probability of distant metastasis.

**FIGURE 6:**
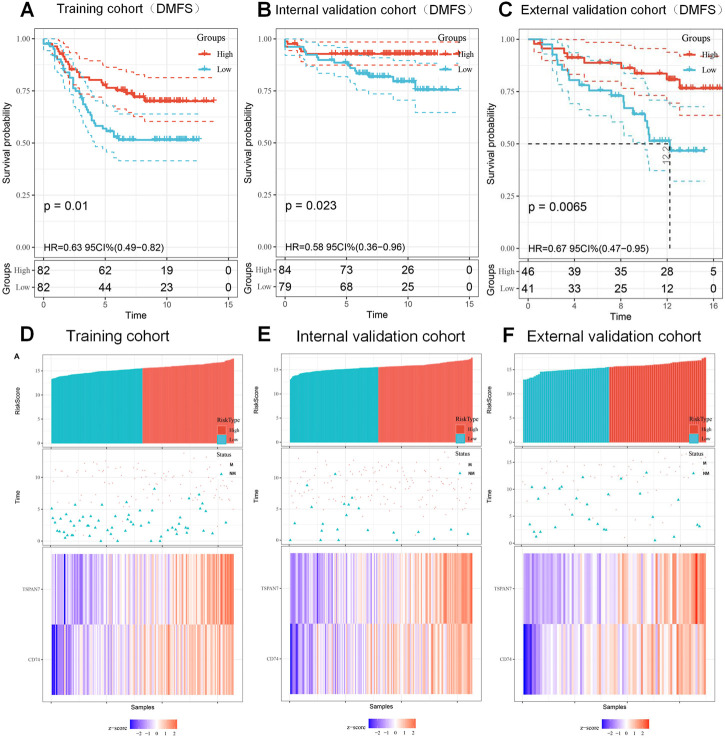
Kaplan-Meier curves of DMFS according to signature and the prognosis of patients with BC. **(A–C)** Kaplan-Meier curves of DMFS based on the signature (15.488) in training cohort **(A)**, internal validation cohort **(B)** and external validation cohort **(C)**. **(D–F)** The distribution of risk score (top), metastatic status (middle) and expression heatmap (bottom) of the two biomarkers in training cohort **(D)**, internal validation cohort **(E)** and external validation cohort **(F)**. M, metastasized; NM, unmetastasized.

To be better applied in clinical diagnosis, a constant cutoff value was determined by the median of the training cohort (15.488). Survival analysis, using the Kaplan-Meier method, indicated that the low score group portends a worse DMFS (HR:0.63; 95%CI:0.49-0.82; *p* = 0.01; Figure 6A). Subsequently, survival analysis was performed twice with the same results in the internal validation cohort (HR:0.58; 95%CI:0.36-0.96; *p* = 0.023; Figure 6B), and the external validation cohort (HR:0.67; 95%CI:0.47-0.95; *p* = 0.0065; Figure 6C), respectively. To individually show the differences between low score and high score groups, we visualized the scores, distant metastasis, and gene expression profiles in the three cohorts (Figures 6D–F). The above results indicated that the signature could predict the risk of metastasis development as an independent risk feature.

### Gene set enrichment analysis

**FIGURE 7:**
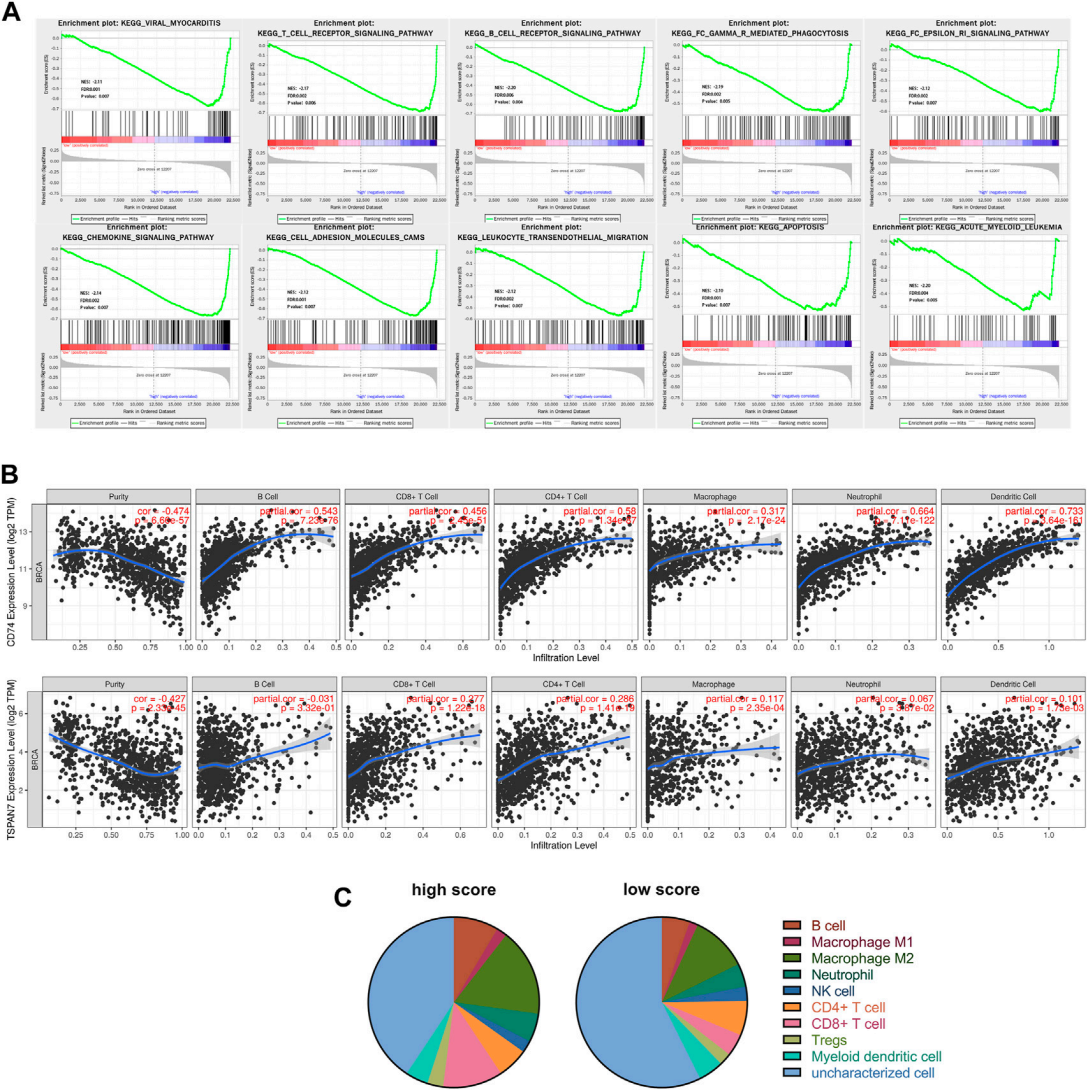
Functional analysis of the CD74 and TSPAN7. **(A)** GSEA identified gene sets significantly enriched in the phenotype of high score patients based on the IIRS. **(B)** Correlations between CD74 or TSPAN7 expression and immune infiltration levels. **(C)** Different levels of immune infiltration were observed between the high score (*n* = 212) and the low score (*n* = 202) groups.

GSEA was performed to analyze the causes of BC metastasis risk difference between high and low score groups. Figure 7A showed that upregulated genes in the high score group were significantly enriched in immune related signal pathways, such as T cell receptor signaling and chemokine signaling pathway, suggesting that our signature may be an immune infection related signature (IIRS).

### Immune infiltration analysis

We further utilized TIMER to analyze the possible correlation between CD74, TSPAN7 expression and levels of immune infiltration in BC (Figure 7B). Both CD74 and TSPAN7 are associated with multiple immune cell infiltration, especially T cells. Therefore, we want to know whether the correlation between the signature and immune infiltration is applicable to all our cohorts. We integrated three cohorts and calculated the fraction of immune cell subsets using quanTIseq. As shown in Figure 7C, the high score group had a higher fraction of B cells (*p* < 0.001), M2 Macrophage (*p* < 0.001), Neutrophil (*p* = 0.009), CD8+T cell (*p* < 0.001), Tregs (*p* < 0.001). On the contrary, the fraction of NK cell (*p* = 0.012) and uncharacterized cells were found to increase significantly in the low score group. It is worth noting that the analysis showed that there was the most significant difference in CD8+T cells between groups.

**FIGURE 8:**
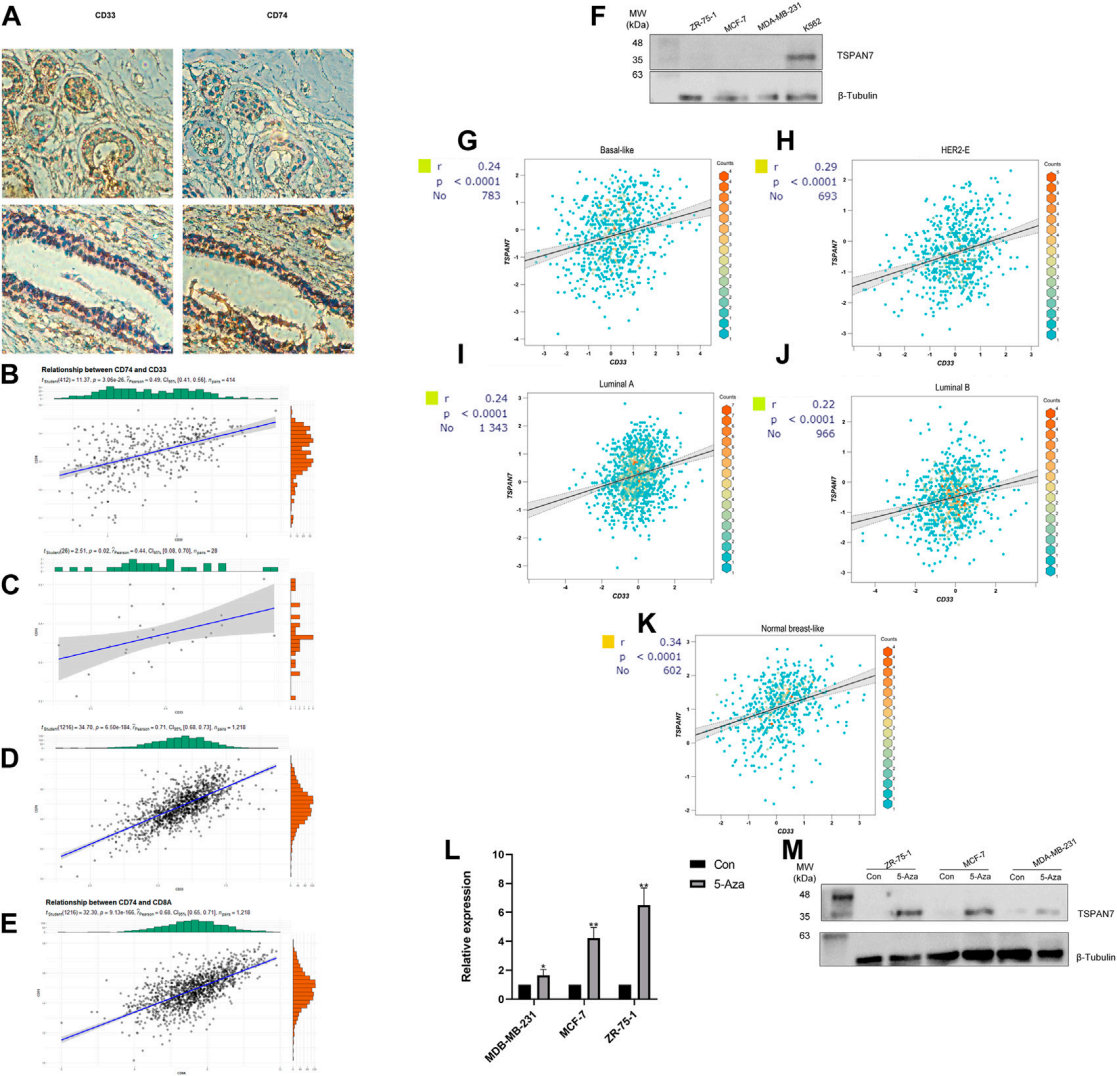
Gene expression and correlation. **(A)** IHC for CD74 and myeloid marker CD33. **(B–D)** Correlation between the expression of CD74 and CD33 in cohorts (Panel B), IHC (Panel C) and TCGA (Panel D) (Pearson correlation coefficient). **(E)** Correlation between the expression of CD74 and CD8A in TCGA. **(F)** Western blot analysis of TSPAN7 in cell lysates of myeloid cells and breast cancer cells. **(G–K)** Correlation between CD33 and TSPAN7 expression of different PAM50 subtypes in bc-GenExMiner. Basal-like subtype **(G)**; HER2-enriched subtype **(H)**; Luminal A subtype **(I)**; Luminal B subtype **(J)**; Nromal-like subtype **(K)**. **(L, M)** TSPAN7 mRNA **(L)** and protein **(M)** expression was upregulated after treatment with the demethylation reagent 5-Aza. *p < 0.05,**p < 0.01, by Student’s t-test.

In view of myeloid cells playing an important role in the recruitment and concentration of CD8^+^ T cells (Jiang et al., 2022), we attempted to evaluate the correlation between myeloid cell levels and the IIRS in BC patients. CD33 is widely used as a marker for tumor infiltrating myeloid cells (Choi et al., 2020; Toor et al., 2021), so we collected FFPEs from 28 consecutive pairs of BC patients and performed IHC for CD74 and CD33, respectively. Interestingly, CD74 protein mainly existed in some CD33^+^ cells (Figure 8A), and was positively correlated with CD33 levels in three cohorts (Figure 8B), IHC (Figure 8C) and TCGA (Figure 8D), respectively. In addition, there is a significant positive correlation between CD74 and CD8 in BC (Figure 8E), which means that CD74^+^ myeloid cells will not affect the recruitment of CD8+T cells.

We detected TSPAN7 protein expression in three common BC cell lines (MCF-7, ZR-75-1, MDA-MB-231) and a myeloid cell line K562 (Figure 8F). TSPAN7 was almost undetectable in BC cell lines, contrary to the myeloid cell line. Importantly, we found that the expression of TSPAN7 was significantly correlated with CD33 in a variety of molecular subtypes, which has been proven to exist mainly in myeloid immune cells (Figures 8G–K). It suggested that the differential expression of TSPAN7 may affect the prognosis by reflecting the infiltration of myeloid cells. However, TSPAN7 expression in BC tissues is lower than that in normal tissues without immune invasion. To explain this paradoxical phenomenon, we hypothesized that TSPAN7 methylation occured and treated three TSPAN7-silenced cells with demethylation agent 5-Aza, respectively. TSPAN7 expression was subsequently restored in all treated BC cells (Figures 8L,M). TSPAN7 methylation in BC cells and myeloid cells infiltration together result in the difference of TSPAN7 expression.

## Construction and assessment of predictive nomogram

**FIGURE 9:**
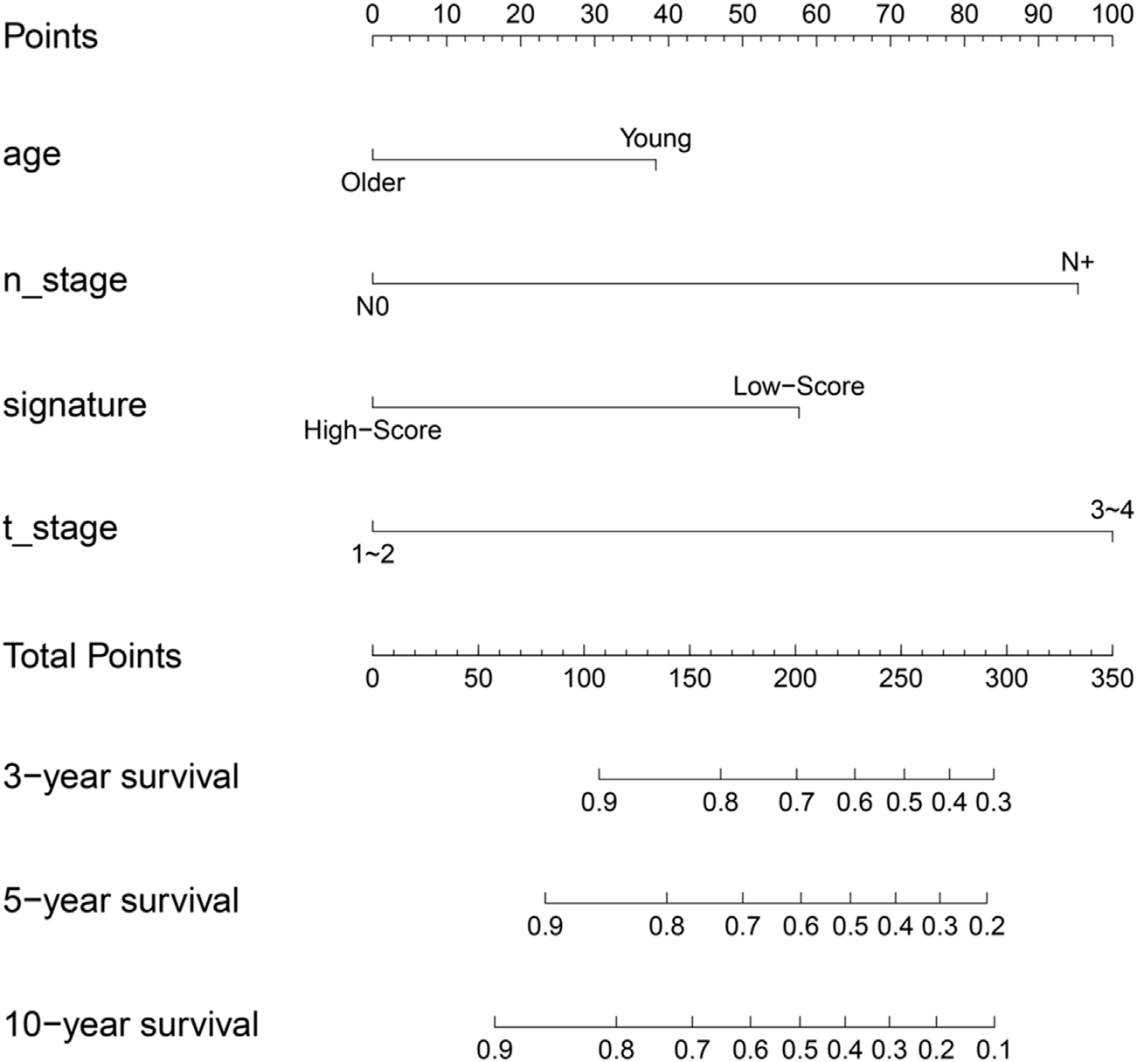
Nomogram to predict for 3-year,5-year and 10-year probabilities of DMFS for breast cancer patients. Age: older: ≥60,young: <60; T_stage: 0: T0; 1: T1; 2: T2; 3: T3; 4: T4; N_stage: 0: N0; N+: N1, N2, N3; signature, IIRS.

In order to increase clinical utility, a predictive nomogram was constructed (Figure 9) based on the risk feature verified by univariate and multivariate Cox analysis, including the IIRS, age, T stage, and N stage (Figures 3C,D). Interestingly, we found that younger patients are at higher risk of metastasis, which was consistent with previous findings (Purushotham et al., 2014).

**FIGURE 10:**
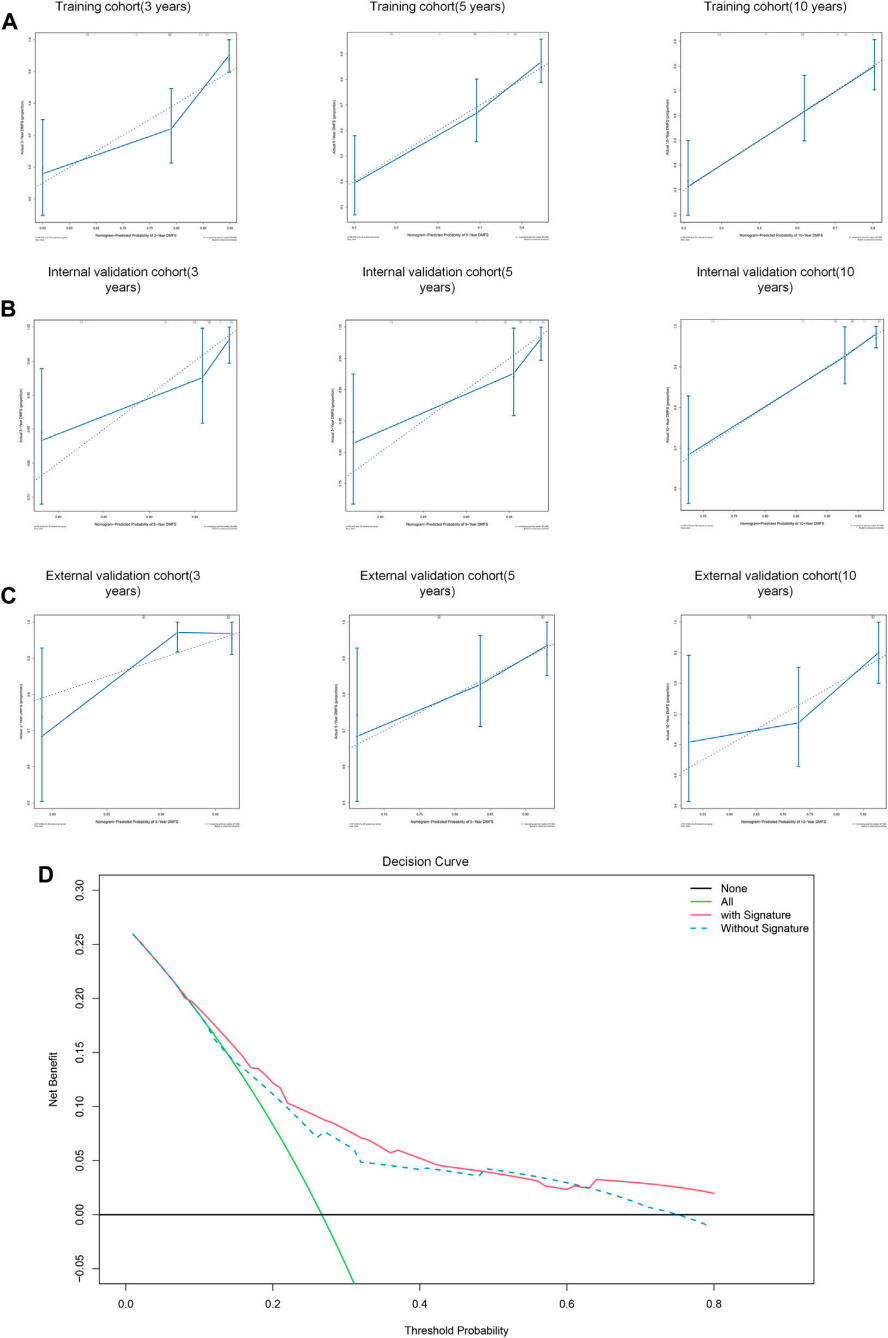
Performance of the nomogram to predict DMFS. **(A–C)** Calibration curves of 3-year, 5-year and 10-year DMFS in training cohort **(A)**, internal validation cohort **(B)**, and external validation cohort **(C)**. **(D)** Decision curve analysis for the nomogram with/without signature.

Subsequently, calibrate curves were plotted to identify the consistency between ideal outcome and actual observation in prediction of 3-,5- 10-year distant metastasis-free survival times. The calibration curves showed good performance in training cohort (Figure 10A), internal validation cohort (Figure 10B), and external validation cohort (Figure 10C), especially for long term survival rate (10-year DMFS).

To assess the predictive performance of this model, the nomogram was internally validated by 1,000 bootstrap resamples in three cohorts. Pleasantly, and surprisingly, the nomogram yielded a C-index of 0.742 (95% CI, 0.715–0.769) for training cohort, 0.801 (95% CI, 0.754 to 0.0.848) for internal validation cohort and 0.695 (95% CI, 0.643 to 0.0.747) for external validation cohort. Therefore, we demonstrated that the nomogram could predict the DMFS of BC patients effectively.

We subsequently performed a decision curve analysis to evaluate the clinical net benefit in predicting the probability of 10- year DMFS. As shown in Figure 10D, if the threshold probability of a patient or doctor is <48% or >63%, using the nomogram with IIRS to predict DMFS adds more benefit than the other without IIRS.

## Discussion

It is well known that almost all metastatic BC has poor overall survival and incurable nature (Gobbini et al., 2018). In recent years, it is gratifying to note that the median survival time after diagnosis of metastatic BC has been increasing due to improved treatment (Mariotto et al., 2017)^.^ Therefore, early assessment and diagnosis of distant metastasis are still meaningful strategies for improving the prognosis of BC patients.

Nowadays multiple biomarkers, such as circulating miRNA (Baldasici et al., 2022), circulating tumor DNA (Page et al., 2021) and circulating tumor cell (Ring et al., 2022), can be used to detect metastasis of BC. What’s more, several serum biomarkers related to metastasis, including soluble POSTN (Jia et al., 2022), PTHrP (Washam et al., 2013), S100P (Peng et al., 2016), have been found. Non-etheless, when these biomarkers increase, it is very likely that BC has undergone distant metastasis (Al-Mahmood et al., 2018). Therefore, improved methods based on gene expression to predict the risk of BC metastasis in advance are needed.

Unfortunately, there is no effective way to achieve this goal. Although PAM50 signature has proven to be able to provide prognostic information from the lymph node metastasis of BC patients, only a few subtypes are associated with metastasis (Tobin et al., 2017; Wang et al., 2019), suggesting that individualized accurate prediction according to PAM50 subtypes is still difficult.

In this study, we sought to identify metastasis-related genes to predict the probability of distant metastasis in BC patients. A total of 62 genes were screened out in GSE184717 and GSE183947.7 genes (CD74,TSPAN7,COL11A1, MMP11, CHRDL1, MELK, PITX1) proved to be associated with DMFS in BC patients, and seven of them (TSPAN7, CD74, MMP11, MELK, COL11A1, CHRDL1, PITX1) were verified by bc-GenExMiner. Two potential biomarkers (CD74, TSPAN7) of metastasis development were used to construct a gene signature by multivariate analysis. The signature proved to be associated with distant metastasis–free survival in three cohort.

The CD74 gene, responsible for producing a protein associate with class II major histocompatibility complex (MHC) is implicated in an effective intratumor immune response (Wang et al., 2017). It also serves as a cell surface receptor for the cytokine macrophage migration inhibitory factor, which may play a pro-oncogenic role in promoting BC cell-stroma interactions (Verjans et al., 2009). Of note, CD74 was observed to be related to triple-negative breast cancer, which is the most aggressive subtype of breast cancer (Tian et al., 2012). The TSPAN7 is a cell-surface protein coding gene, and the coding protein of which plays a role in the regulation of cell development, activation, growth and motility (Perot and Ménager, 2020). For this reason, TSPAN7 is also known as CD231. Our study is the first to link CD74 and TSPAN7 expression with distant metastasis–free survival in breast cancer, highlighting gene signature based on CD74 and TSPAN7 as a predictor of metastasis development in BC, with a strong effect on patients’ distant metastasis–free survival.

In past studies, multiple prognostic models have been constructed because of the differential expression of CD74 (Wang et al., 2020) or TSPAN7 (Wu et al., 2020) between tumor and normal tissues. However, the reason for this difference is still unclear. As a result, much effort has been expended in trying to explore differential expression of CD74 or TSPAN7 in cancer cell strains, ignoring the influence of immune infiltration (Greenwood et al., 2012; Wang et al., 2018; Qi et al., 2020; Yu et al., 2021). Notably, heterogeneity of CD74 expression has been confirmed in tumor tissues (Richard et al., 2014), indicating that characterization of immune landscape cannot be discounted. In this study, GSEA revealed a statistical enrichment of immune-related signaling pathways in the high score group. Thus we speculated that the signature we constructed may reveal the levels of immune infiltration. The results of TIMER database confirmed this conjecture. CD74 and TSPAN7 expression was negatively correlated with tumor purity while positively correlated with the level of multiple immune cells. Furthermore, the fraction of immune cells in high score group was significantly higher than that in low score group, especially CT8+ T cells. According to the results presented above, we identified a novel cause of differential expression of CD74 and TSPAN7. The levels of CD74 and TSPAN7 reflected the ability of immune cells to infiltrate tumors. To the best of our knowledge, this is the first study to show that high CD74 and TSPAN7 expression is associated with tumor-infiltrating immune cells. Further studies on Intra-tumoral heterogeneity are warranted, in order to analyze the relationship between survival time and the level of immune cell infiltration.

Our study has some potential limitations. First, pathologic features were unavailable, so the correlation between our signature and pathological features could not be evaluated. Second, quanTIseq is a prediction tool of immune cell fraction based on deconvolution algorithm, as a result, it will predict a minimal amount of immune cells even though they are absent (Sturm et al., 2019).

In conclusion, we identified seven genes related to distant metastasis–free survival, namely, TSPAN7, CD74, MMP11, MELK, COL11A1, CHRDL1, PITX1, and a signature based on CD74 and TSPAN7 expression that may have potential of predicting DMFS. We found that methylation of TSPAN7 in BC cells inhibits the recruitment of CD74 positive immune cells, which may be associated with a lower risk of metastasis. The present study was the first to propose that high CD74 expression may be derived from tumor-infiltrating immune cells. Given the favorable discrimination of the signature, we developed a nomogram for clinical applications. This is the first nomogram based on gene signature that can be used to facilitate the individualized prediction of DMFS in BC patients.

## Data Availability

The original contributions presented in the study are included in the article/supplementary material, further inquiries can be directed to the corresponding authors.
